# Analytical Methodology for Sensitive Detection of the Antidepressant Drug Duloxetine in Urine Samples Using Riboflavin-Coated Fe_3_O_4_ Magnetic Particles

**DOI:** 10.1021/acsomega.5c12023

**Published:** 2026-08-10

**Authors:** Rihab Gargouri, Halil İbrahim Ulusoy, Ümmügülsüm Polat, Erkan Yılmaz, Gökhan Sarp, Ramzi Maalej, Kamel Damak

**Affiliations:** † LaMaCoP, Faculty of Sciences of Sfax, 479733 University of Sfax, Sfax 3018, Tunisia; ‡ Department of Analytical Chemistry, Faculty of Pharmacy, Sivas Cumhuriyet University, Sivas 58140, Turkiye; § Department of Analytical Chemistry, Faculty of Pharmacy, Erciyes University, Kayseri 38280, Turkiye

## Abstract

Antidepressant drugs are widely used and effective in treating mood disorders. Monitoring low concentrations of these drugs in biological samples such as solid tissues and biofluids with high sensitivity is challenging for conventional analysis methods. The present study aims to determine duloxetine molecules at trace levels in urine samples by using a simple sample preparation method and a high-performance liquid chromatography coupled with diode array detection (HPLC-DAD) system. This study presents the development of a novel separation and preconcentration method for the trace-level determination of duloxetine using HPLC-DAD following magnetic solid-phase extraction (MSPE). For this purpose, tetraethyl orthosilicate (TEOS)-coated magnetic nanoparticles functionalized with riboflavin (Fe_3_O_4_@TEOS-riboflavin) were synthesized and thoroughly characterized by scanning electron microscopy (SEM), X-ray diffraction (XRD), and Fourier-transform infrared (FTIR) spectroscopy. Following MSPE, duloxetine exhibited a linear response over the range of 10.00–800.00 ng·mL^–1^ (R^2^: 0.9942). The limits of detection (LOD) and quantification (LOQ) were determined to be 3.28 and 9.60 ng·mL^–1^, respectively. Relative standard deviations (RSDs) were below 5.1% for triplicate analyses of model solutions containing 100 ng·mL^–1^ of duloxetine. Moreover, the developed method was successfully applied to both synthetic and healthy human urine samples, providing quantitative results in recovery studies. The recovery values for synthetic and human urine samples ranged from 98.1 to 102.2%, indicating that the combination of MSPE with HPLC-DAD provides a reliable approach for the accurate determination of duloxetine concentrations in urine.

## Graphical abstract

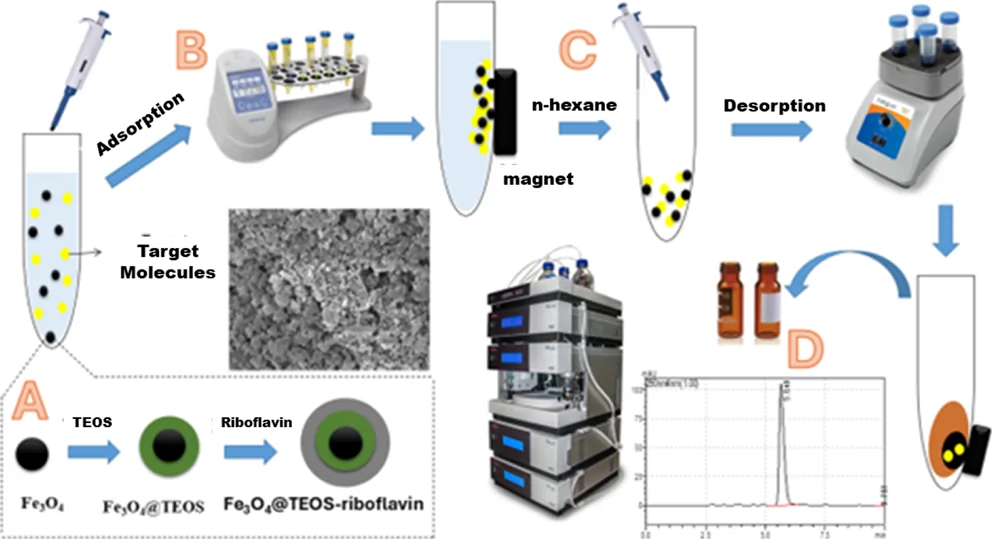

## Introduction

As a widespread mental health disorder, depression represents a significant global healthcare concern. Advances in antidepressant pharmacotherapy have expanded treatment options considerably, beginning with the development of monoamine oxidase inhibitors and tricyclic antidepressants.^1^ Among the available agents, serotonin–norepinephrine reuptake inhibitors (SNRIs) have gained prominence as first-line medications for a broad spectrum of psychiatric and neurological disorders, including anxiety disorders, eating disturbances, post-traumatic stress disord (PTSD), pain-related conditions, and premenstrual dysphoric disorder.^2^ Furthermore, major depressive disorder (MDD) together with generalized anxiety disorder (GAD) accounts for a large proportion of the global mental health burden and is associated with impaired cognition, reduced occupational functioning, and diminished quality of life.^3^

Duloxetine (DLX) ((+)-(S)-*N*-methyl-3-(1-naphthyloxy)-3­(thiophen-2-yl)-propan-1-amine hydrochloride) is a SNRI group drug and widely prescripted under various trade names for the treatment of MDD and GAD.^4–8^ Although its clinical efficacy and tolerability in the management of MDD and GAD have been confirmed,^9–12^ DLX therapy is often accompanied by adverse effects, including cardiovascular complications, overdose-related toxicity, and a heightened risk of suicidal ideation—particularly in pediatric and adolescent populations.^13–15^ These concerns necessitate the implementation of therapeutic drug monitoring (TDM) strategies to ensure plasma concentrations remain within the therapeutic range, thereby minimizing toxicity, therapeutic failure, or patient noncompliance.^16,17^ Biological matrices such as plasma, urine, and saliva are inherently complex and contain a wide range of endogenous substances, which can interfere with the detection of antidepressants present at trace levels.^18^ Accurate quantification in such matrices thus requires effective sample preparation techniques that enable both matrix cleanup and analyte enrichment.

Several analytical platforms have been developed for the trace-level determination of DLX in biological and pharmaceutical media, including liquid chromatography–mass spectrometry (LC–MS),^19,20^ tandem mass spectrometry (LC–MS/MS),^21,22^ gas chromatography with nitrogen–phosphorus detection,^23^ high-performance liquid chromatography with UV and fluorescence detection (HPLC-UV, HPLC-FLD),^24,25^ and ultrahigh-performance liquid chromatography coupled with tandem mass spectrometry (UHPLC–MS/MS).^26^ However, these techniques require prior sample pretreatment to isolate DLX from interfering with matrix components. Traditional methods such as liquid–liquid extraction (LLE)^27^ and solid-phase extraction (SPE)^24^ have limitations, including labor-intensive protocols, extended processing times, and reliance on large volumes of toxic organic solvents.^28^ While SPE offers advantages in terms of selectivity and solvent efficiency,^29^ recent efforts have focused on the development of novel, green extraction technologies employing reusable sorbents to reduce time, cost, and environmental burden.^30^ Magnetic nanoparticles have emerged as highly promising materials for such applications due to their high surface area, ease of manipulation, and rapid phase separation.^31^ The use of magnetic solid-phase extraction (MSPE), first reported for antidepressants by Chen et al. in 2014, offers notable advantages over conventional sorbents.^32^ Nevertheless, unmodified magnetic nanoparticles are susceptible to oxidation and require surface functionalization using silica, carbon, polymers, or surfactants to enhance stability and specificity.^33,34^

In the present study, riboflavin-functionalized Fe_3_O_4_-TEOS nanoparticles (Fe_3_O_4_@TEOS-riboflavin) were synthesized and applied as sorbents in an MSPE protocol for the extraction and preconcentration of DLX from urine samples. Coupled with HPLC-DAD detection, this method provides a simple, sensitive, and environmentally sustainable approach to therapeutic drug monitoring and toxicological analysis.

## Materials and Methods

### Instrumentation

Material characterization of the Fe_3_O_4_@TEOS-riboflavin core–shell magnetic nanostructure was performed using XRD (Bruker AXS D8, Japan), FTIR spectroscopy (PerkinElmer, USA), and SEM–EDX analysis (Zeiss Gemini 500). Duloxetine analyses were conducted with a Shimadzu high-performance liquid chromatography system (Kyoto, Japan) comprising an LC20-AD pump, SPD-M20 DAD detector, DGU-20A vacuum degasser, and CTO-10 column thermostat. Data collection, system control, and chromatographic evaluation were achieved using LC Solutions 2.0 software. A pH-2005 instrument from JP Selecta (Barcelona, Spain) was utilized for pH determination. Sample mixing steps were carried out with a Jeotech vortex mixer and a WhyShake SHO-2D orbital shaker. Ultrapure water generated by an MP Minipure Dest Up system (MES, Ankara, Türkiye), exhibiting a resistivity of 18.2 MΩ·cm at 25 °C, was employed in the preparation of all aqueous solutions.

### Chemicals and Reagents

All analytical reagents employed in the experiments were supplied by Merck and Sigma-Aldrich companies and were used without further purification: FeCl_3_·6H_2_O (>99.0%, Merck, CAS Number: 10025-77-1), FeSO_4_·4H_2_O (>99.0%, Merck, CAS Number: 7782-63-0), tetraethoxysilane (TEOS) (>99.0%, Merck, CAS Number: 78-10-4), and riboflavin (>99.0%, Merck, CAS Number: 83-88-5). The eluent phase for chromatographic measurements was prepared using HPLC-grade acetonitrile (ACN) (>99.0%, Merck, CAS Number: 75-05-8) and methanol (MeOH) (>99.0%, Merck, CAS Number: 67-56-1) solvents for HPLC-DAD analyses. A stock solution of 1000 μg mL^–1^ of duloxetine (CAS Number: 136434-34-9) was prepared in methanol, and the working standard solutions used in optimization steps were prepared by appropriate serial dilution of the stock solution with methanol and stored at +5 °C in a dark glass bottle. The pH of the working solutions was adjusted using a Britton–Robinson (BR) buffer series covering a pH range from 1.0 to 11.0, prepared from a stock BR solution containing 0.02 M concentrations of H_3_BO_3_ (>99.0%, Merck, CAS Number: 10043-35-3), H_3_PO_4_ (>99.0%, Merck, CAS Number: 7664-38-2), and CH_3_COOH (>99.0%, Merck, CAS Number: 64-19-7) acids.

### Chromatographic Conditions

An isocratic HPLC method was employed using a ternary mobile phase consisting of acetate buffer (50 mM, pH 5.0), methanol, and acetonitrile mixed in a 30:50:20 (v/v/v) ratio. The eluent was delivered at 1.0 mL min^–1^. Chromatographic monitoring was performed at 290 nm using a DAD system, and the column compartment temperature was fixed at 40 °C. All experiments were conducted on a C18 Inertsil ODS-3 column supplied by GL Sciences (250 mm × 4.6 mm, 5 μm). The injection volume was set to 10 μL for every measurement. Before chromatographic use, the mobile phase was filtered through a 0.45 μm membrane and degassed to remove dissolved gases. As illustrated in Figure 1, duloxetine exhibited a retention time of 5.6 min under the optimized conditions. Analysis of MSPE-treated model solutions containing 100 ng mL^–1^ DLX yielded a distinct analyte peak without observable matrix interference around the retention region of interest.

**Figure 1:**
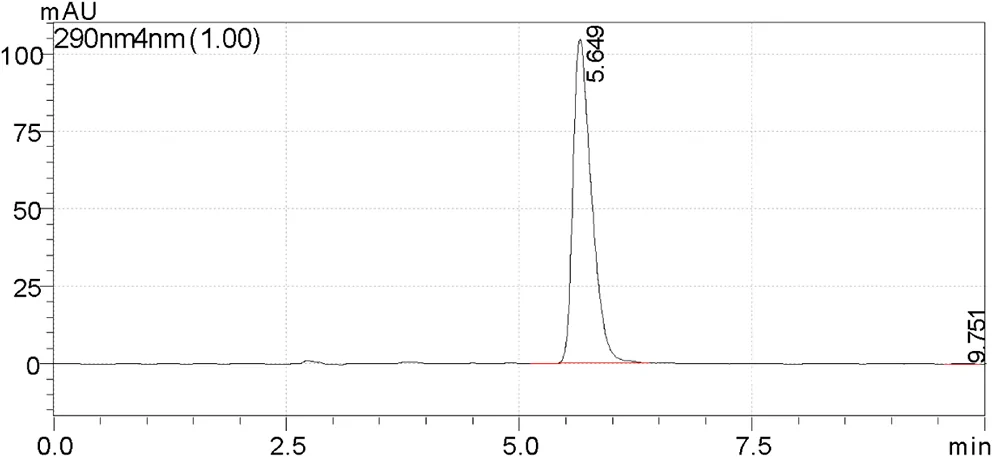
Chromatogram of duloxetine (DLX) molecule after magnetic solid-phase extraction (MSPE) reported at 290 nm (100 ng mL^–1^).

### Preparation of Magnetic Sorbent (Fe_3_O_4_@TEOS-riboflavin)

The synthesis of magnetic nanoparticles followed a well-established protocol from our earlier work.^35,36^ To prepare the core magnetite, 9.72 g of FeCl_3_·6H_2_O and 3.78 g of FeSO_4_·4H_2_O (1:2 molar ratio) were dissolved in a 0.01 M HCl solution (50 mL). This mixture was combined with 50 mL of 50% methanol and heated to 85 °C under constant stirring at 600 rpm. Precipitation of Fe_3_O_4_ was induced by the slow, dropwise addition of 150 mL of 1.5 M NaOH into the swirling solution. An external magnetic field was applied to isolate the resulting black magnetite particles, which were subsequently washed with distilled water and alcohol before being dried at 60 °C for 6 h.

For the surface modification stage, a 2.5 g portion of these Fe_3_O_4_ cores was suspended in a mixture of 50% methanol (100 mL), aqueous ammonia (3 mL), and TEOS (2.5 mL) to drive the silanization process. The functionalized Fe_3_O_4_@SiO_2_ spheres were magnetically collected, rinsed thoroughly using ethanol, and left to dry at room temperature. These silica-coated particles were then introduced into a 1.5 M NaOH solution (50 mL) containing 200 mg of dissolved riboflavin. This mixture was stirred at 500 rpm under a protective nitrogen atmosphere for 4 h at 45–50 °C, followed by an additional 6 h of aging at room temperature. The final Fe_3_O_4_@TEOS-riboflavin nanocomposite was recovered, washed repeatedly with a water–methanol blend, dried overnight, and ultimately ground and sieved for size uniformity.

### The Proposed MSPE-Coupled HPLC-DAD Method

To optimize the MSPE conditions, a 50 mg portion of the Fe_3_O_4_@TEOS-riboflavin magnetic adsorbent was introduced into 50 mL Falcon tubes. Each tube was then treated with 2 mL of a sample solution containing DLX at concentrations ranging from 10.00 to 800.00 ng mL^–1^. Method parameters were controlled by adjusting the sample pH to 10.0 via the addition of 1.0 mL Britton–Robinson (BR) buffer, after which the mixture was diluted to a final volume of 50 mL using ultrapure water. The tightly capped tubes were agitated on a rotating shaker (50 rpm) for 30 min to ensure complete adsorption of DLX onto the magnetic core–shell nanostructures.

Once the adsorption step concluded, an external magnet was utilized to achieve rapid phase separation between the nanoparticles and the aqueous medium. The supernatant was decanted, and the target analyte was desorbed from the isolated solid phase by adding 500 μL of *n*-hexane, followed by 40 s of vigorous vortexing. The magnetic sorbent was isolated once more using the magnet, and the collected eluent was passed through a 0.45 μm PTFE syringe filter directly into autosampler vials for subsequent HPLC-DAD quantification. Regeneration of the magnetic particles was performed by rinsing with 2 mL of methanol after each run to confirm reusability. A schematic representation of the entire MSPE-HPLC-DAD workflow is depicted in Figure 2.

**Figure 2:**
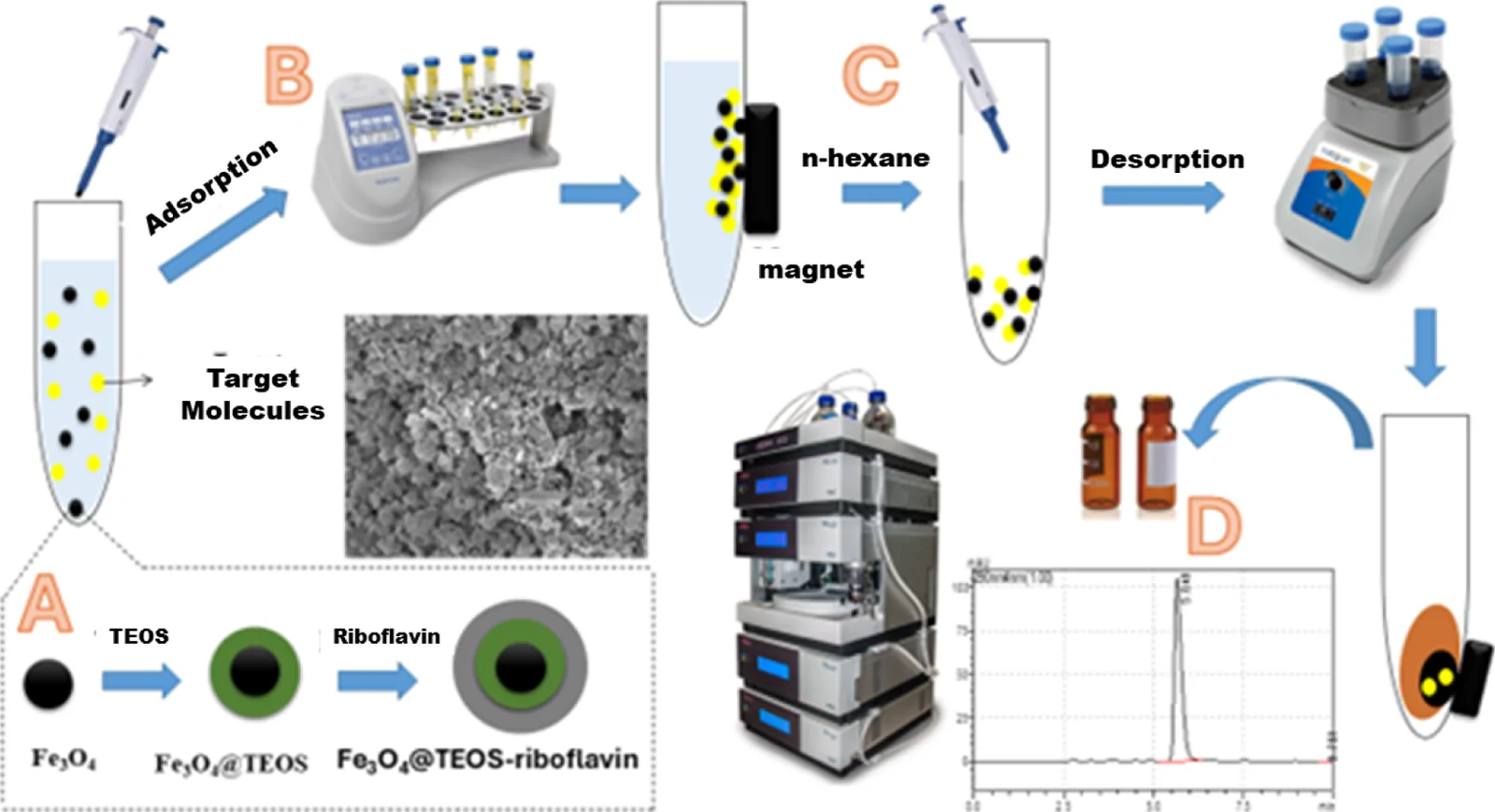
Main steps of the proposed method: A: synthesis of magnetic sorbent, B: adsorption of target molecules on magnetic sorbent, C: desorption of retained molecules by n-hexane, and D: chromatographic analysis of duloxetine molecules.

## Results and Discussion

### Characterization of Magnetic Nanoparticles

The core–shell Fe_3_O_4_ was modified with TEOS and riboflavin, and characterized by using XRD, FTIR, and SEM techniques.

### XRD Analysis

For the XRD spectrum of Fe_3_O_4_ NPs (Figure 3), the 2θ values of 31.0°, 35.3°, 43.8°, 52.6°, and 57.8° correspond to the (220), (311), (400), (422), and (511) planes of the magnetite Fe_3_O_4_ NP phase. The peaks obtained for Fe_3_O_4_ NPs are consistent with the cubic structure of magnetite Fe_3_O_4_ NPs. The characteristic XRD diffraction peaks for Fe_3_O_4_ modified TEOS-riboflavin NPs are at 2θ = 27.5°, 31.87°, 45.6°, 56.7°, 62.8°, 66.8°, and 75.8°. The characteristic peaks of riboflavin are at 2θ = 16.2°, 20.4°, 27.1°, and 30.1°, and these XRD diffraction peaks match those reported in the literature.^37^

**Figure 3:**
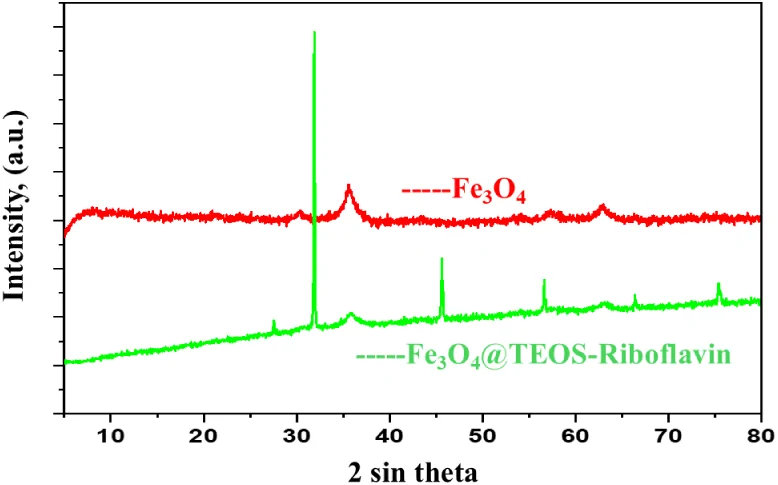
Structural characterization of modified and unmodified nanoparticles using X-ray diffraction (XRD) technique.

### FTIR Spectroscopy Analysis

The FTIR spectra of Fe_3_O_4_ and Fe_3_O_4_@TEOS-riboflavin materials were recorded in the wavenumber range of 4000–450 cm^–1^ (Figure 4). A particularly strong peak around 568 cm^–1^ corresponds to Fe–O vibration modes. The peaks of Fe_3_O_4_ include a strong C=C stretching at 1620–1610 cm^–1^, and a strong C–H stretching vibrations peak around 2934 cm^–1^. The presence of this band in the bare Fe_3_O_4_ spectrum suggests that it may originate from residual organic species associated with the synthesis process. The band observed around 1620 cm^–1^ can be attributed to C=C and N–H related vibrations of riboflavin; however, this region may also include contributions from other surface functionalities. Therefore, the assignment is supported by overall spectral changes rather than a single peak. Although the C=O stretching band of the isoalloxazine ring is typically expected around 1700 cm^–1^, in the present spectrum this region appears less distinct, possibly due to overlapping with adjacent bands or reduced signal intensity after surface immobilization. A broad absorption band in the 3100–3300 cm^–1^ region was also observed and can be attributed to N–H stretching vibrations of riboflavin, although partial overlap with O–H groups and hydrogen bonding effects may contribute to band broadening.

**Figure 4:**
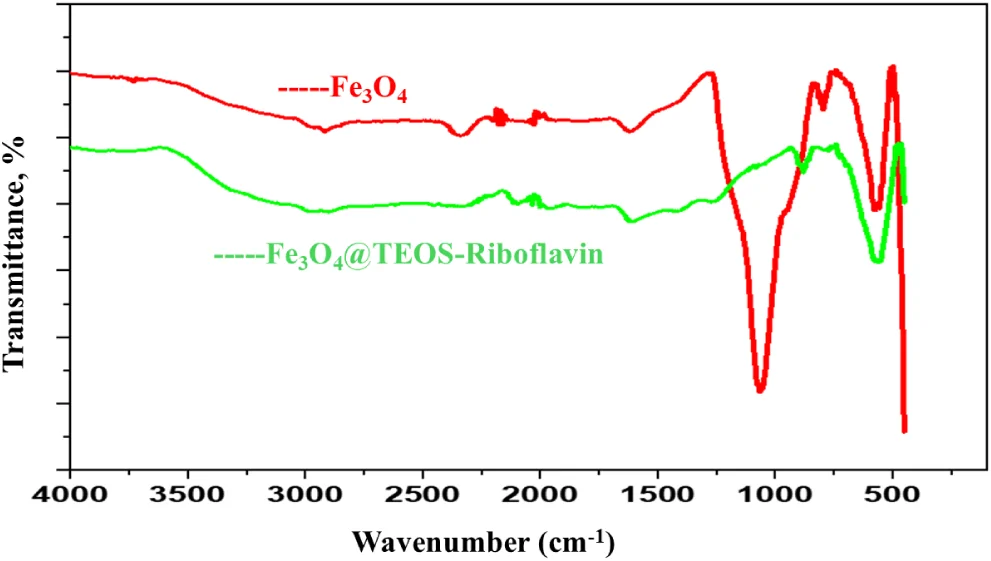
Structural characterization of the used magnetic sorbent nanoparticles using Fourier transform infrared spectroscopy (FTIR).

Additionally, a notable band appeared near 1100 cm^–1^ in the Fe_3_O_4_ spectrum. The disappearance of the ∼1100 cm^–1^ band after modification is attributed not only to surface transformation but also to the formation of the silica shell and overlapping Si–O–Si stretching vibrations around 1080 cm^–1^, which may mask residual iron oxide-related signals. While not typically assigned to the magnetite phase, this band has been reported to originate from hydrated iron oxide species such as lepidocrocite (γ-FeO­(OH)), hydrohematite, or ferrihydrite, possibly formed due to structural water or hydroxylation during the aqueous synthesis process. These observations are supported by the spectral atlas published by Dufour et al.^38^ Riboflavin exhibits characteristic peaks corresponding to N–H, N=C, C–C, C=C, and −OH functional groups. When the FTIR spectrum of the final product is investigated, significant shifts in the spectrum, along with variations in peak intensity levels and forms, are observed. The peaks are observed at 2300, 1620, 1257, 873, and 566 cm^–1^, respectively. The weak band near 2340 cm^–1^ was attributed to atmospheric CO_2_ background and was excluded from structural interpretation. The stretching band in the range of 2350–2280 cm^–1^ is attributed to O=C=O, while medium-strength N–H bending and C–C stretching peaks are observed in the range of 1650–1580 cm^–1^. Importantly, the peak near 1100 cm^–1^ observed in the unmodified Fe_3_O_4_ spectrum disappears after modification with TEOS and riboflavin. This is not due to a simple peak loss but rather reflects a chemical transformation. Upon surface modification, the silica shell introduces new vibrational modes, particularly the symmetric and asymmetric stretching vibrations of Si–O–Si bonds, which appear clearly around 880–1100 cm^–1^. The band observed near ∼880 cm^–1^ is attributed to the symmetric stretching of siloxane (Si–O–Si) bridges, indicating the formation of a silica network on the nanoparticle surface. As a result, the spectral profile changes due to both the addition of new functional groups and the overlapping of vibrational bands, which alter the peak shape and intensity rather than eliminating it.

### SEM Analysis

To characterize the morphological features and particle size of the synthesized magnetic nanoparticles (Fe_3_O_4_ and Fe_3_O_4_@TEOS-riboflavin), SEM analyses were performed at magnification levels of 10,000× and 30,000×, as shown in Figure 5a and b, respectively. The SEM images confirmed the successful formation of Fe_3_O_4_ particles and the effective incorporation of the TEOS layer onto the Fe_3_O_4_ surface. EDX analysis was employed to further verify surface modifications. The resulting spectra, displayed in Figure 5c and d, illustrate the elemental composition of the nanoparticles before and after chemical modification. The unmodified Fe_3_O_4_ nanoparticles exhibited a layered, spherical morphology with a notably high surface area, although the particles lacked perfect sphericity. In contrast, the Fe_3_O_4_@TEOS-riboflavin nanostructures showed a significant morphological transformation, with the magnetic Fe_3_O_4_ core uniformly coated by the riboflavin-modified TEOS shell across the surface.

**Figure 5:**
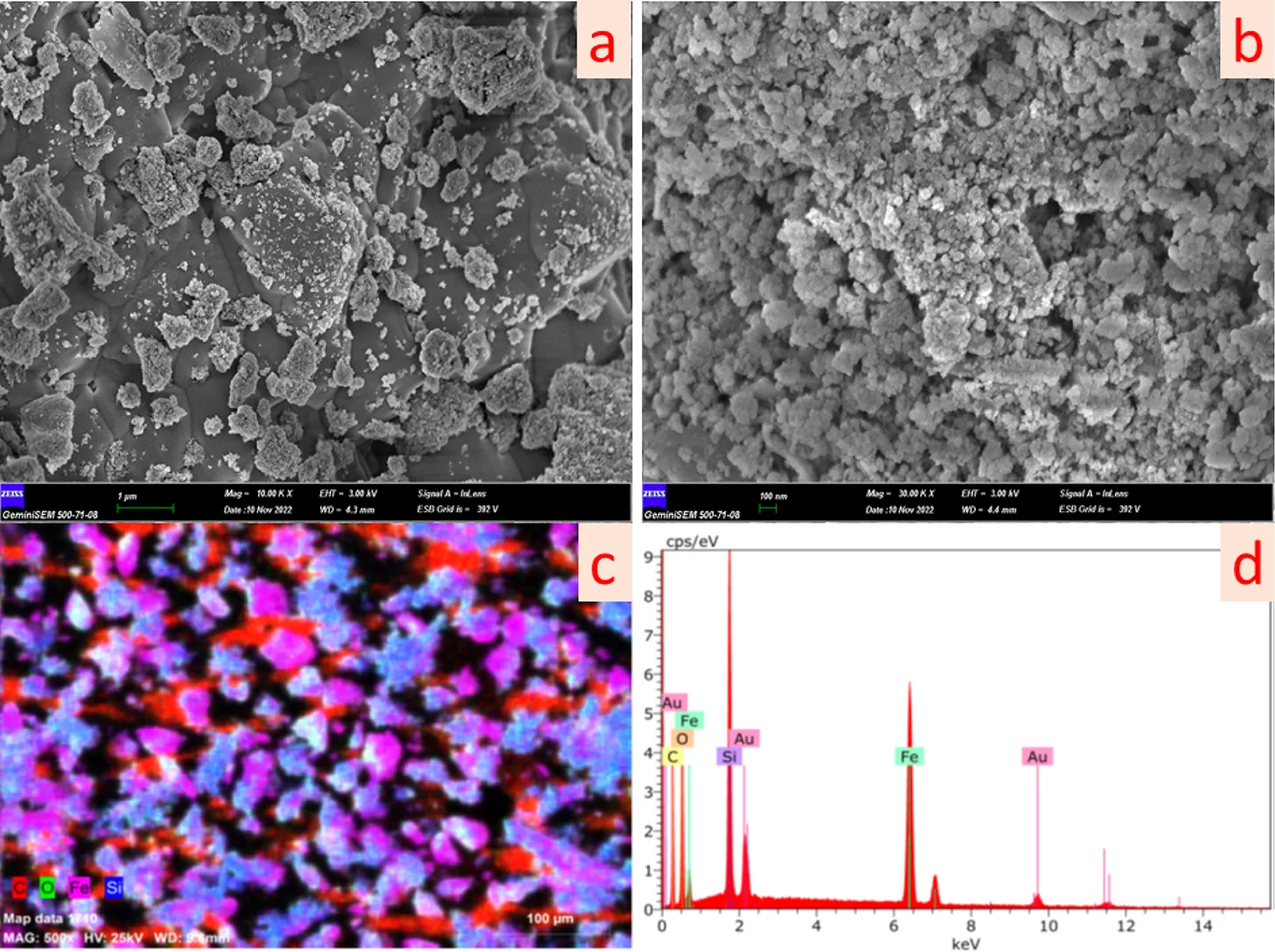
Scanning electron microscopy (SEM) images of Fe_3_O_4_ (a), Fe_3_O_4_@TEOS-riboflavin (b), and SEM-EDX mapping images (c) and elemental analysis (d) of Fe_3_O_4_@TEOS-riboflavin nanoparticles.

As depicted in the mapping mode image (Figure 4c), the modifying agent appeared to be homogeneously distributed throughout the particle surface. The presence of silicon (Si) and carbon (C) atoms—attributed to the TEOS and riboflavin components, respectively—was clearly detected throughout the nanoparticle structure. This elemental distribution was further confirmed by the EDX spectra shown in Figure 5d, validating the successful functionalization of the Fe_3_O_4_ surface with TEOS and riboflavin.

### Optimization of the MSPE Procedure

Herein, a novel analytical approach is described for the determination of DLX in human urine matrices. This methodology integrates an efficient enrichment and isolation protocol via MSPE coupled to an HPLC-DAD system. The primary objective is to selectively isolate target analytes from complex matrix interference by maximizing their retention parameters on the magnetic sorbent. Following matrix separation, quantitative elution of the captured compounds into appropriate organic solvents was successfully completed. The operational framework of the MSPE technique fundamentally relies on two interconnected steps: analyte adsorption and desorption. To yield peak extraction recovery and enhance the overall method sensitivity, each phase underwent systematic and independent optimization.

A classical “one-variable-at-a-time” approach was implemented to evaluate and refine individual experimental parameters governing the extraction performance of the magnetic nanostructures. These key variables included sample pH, contact duration, eluent chemistry, desorption volume, and vortex time. Initial efforts focused on maximizing adsorption behaviors by adjusting both the specimen pH and the contact interval. In the sequential phase, the isolation workflow was finalized by maximizing the recovery parameters during the desorption of duloxetine molecules from the functionalized surface of the magnetic nanosorbent.

### Effect of pH on Adsorption Efficiency

One of the most critical factors in the extraction process is the pH of the sample solution, as it influences the adsorption of target analytes onto the solid phase, dictates the nature of interactions with the magnetic sorbent, and affects the surface charge of the sorbent. Adjusting the pH to an appropriate level is the first optimization parameter to ensure efficient transfer of target molecules to the solid surface. To investigate this, 1 mL of BR buffer with a pH range of 2.0–11.0 was added to a series of tubes containing the magnetic sorbent and duloxetine. Subsequently, the liquid phase samples were collected using a syringe, filtered through a 0.45 μm PTFE membrane, and transferred to HPLC vials for analysis. As shown in Figure 6, the magnetic sorbents exhibited the highest affinity for duloxetine at pH 10.0. This observation corresponds with the p*K*_a_ value of duloxetine (9.7),^41^ as the molecule predominantly exists in its unprotonated form above this pH, facilitating stronger interactions with the magnetic particle surface. Therefore, pH 10.0 was selected as the optimum for subsequent MSPE experiments.

**Figure 6:**
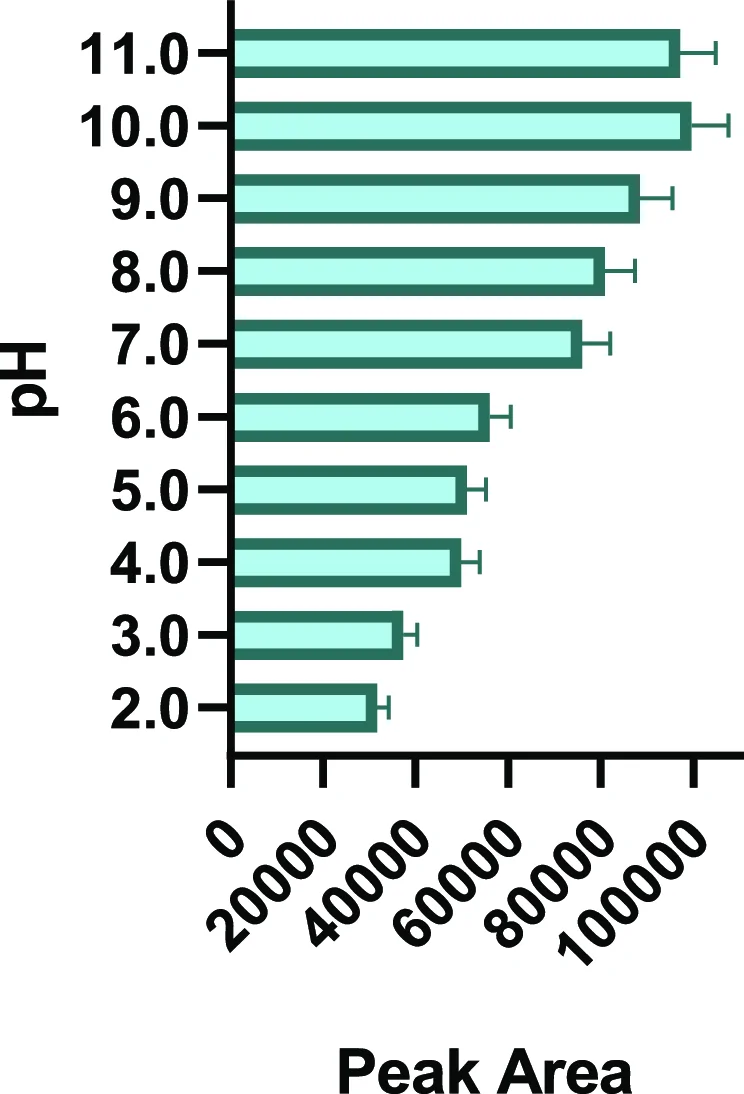
Effect of pH on the magnetic solid-phase extraction (MSPE) efficiency (N:3) using model solutions with 250 ng mL^–1^ of duloxetine (DLX).

### Optimization of Adsorption Time in MSPE

Preconcentration procedures aim to analyze the samples in the shortest possible period. The analyte partition coefficient between the liquid sample matrix and the magnetic nanoparticle controls the equilibrium mechanism used to adsorb drug molecules on the magnetic nanoparticle surfaces. DLX molecules must remain on the surface of the magnetic nanoparticles for a sufficient time. The dispersion of the sorbent and the extraction efficiency depend upon the adsorption, which is expressed as the extraction time. The initial stage of extraction (adsorption) was performed by using a rotating shaker at 50 rpm to increase contact between the adsorbent and the sample solution. At room temperature, model solutions including target molecules were placed on the orbital shaker, and the adsorption time was studied in the range of 0–90 min. The quantitative adsorption of duloxetine particles ended within 30 min at a shaking rate of 50 rpm (Figure 7). There was no significant change in the extraction efficiency beyond this point; it even slightly decreased and remained constant due to the removal of weakly binding compounds. Therefore, in the subsequent experiments, the samples were left on a rotating shaker for 30 min.

**Figure 7:**
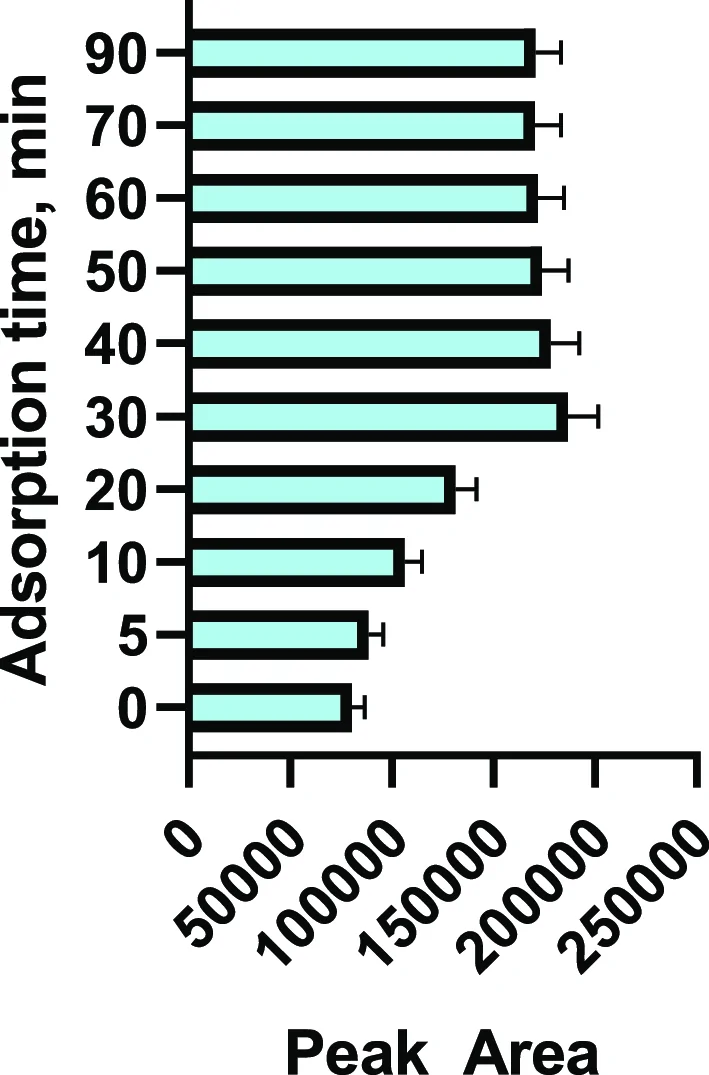
Effect of adsorption time on magnetic solid-phase extraction by means of model solutions with 250 ng mL^–1^ of duloxetine (DLX) (N:3).

### Effect of Desorption Conditions on MSPE

To maximize the intensity of the analytical signal, optimizing both the chemical nature and the total volume of the eluting agent is imperative for releasing trapped analytes from the sorbent matrix. Attaining an optimal preconcentration factor (PF) fundamentally requires an eluent that facilitates swift and quantitative desorption using minimal volumes. To evaluate the ideal desorption medium, a series of organic and aqueous solutions (1 mL each) were systematically screened, including methanol, ethanol, acetonitrile, distilled water, acetone, 2-propanol, *n*-hexane, an acetonitrile-methanol blend (1:1, v/v), 50% methanol, and a pH 10 buffer. A key consideration during selection was the chemical compatibility of the eluent with the downstream HPLC mobile phase.

Experimental outcomes demonstrated that the most prominent chromatographic response, in terms of peak area, was achieved when utilizing *n*-hexane for the back-extraction of duloxetine, as detailed in Figure 8. Because the target analyte exhibits poor desorption behavior in highly polar environments, aqueous options like distilled water and buffered media yielded negligible recoveries. Consequently, *n*-hexane was chosen as the standard desorption solvent for all of the subsequent extraction protocols.

**Figure 8:**
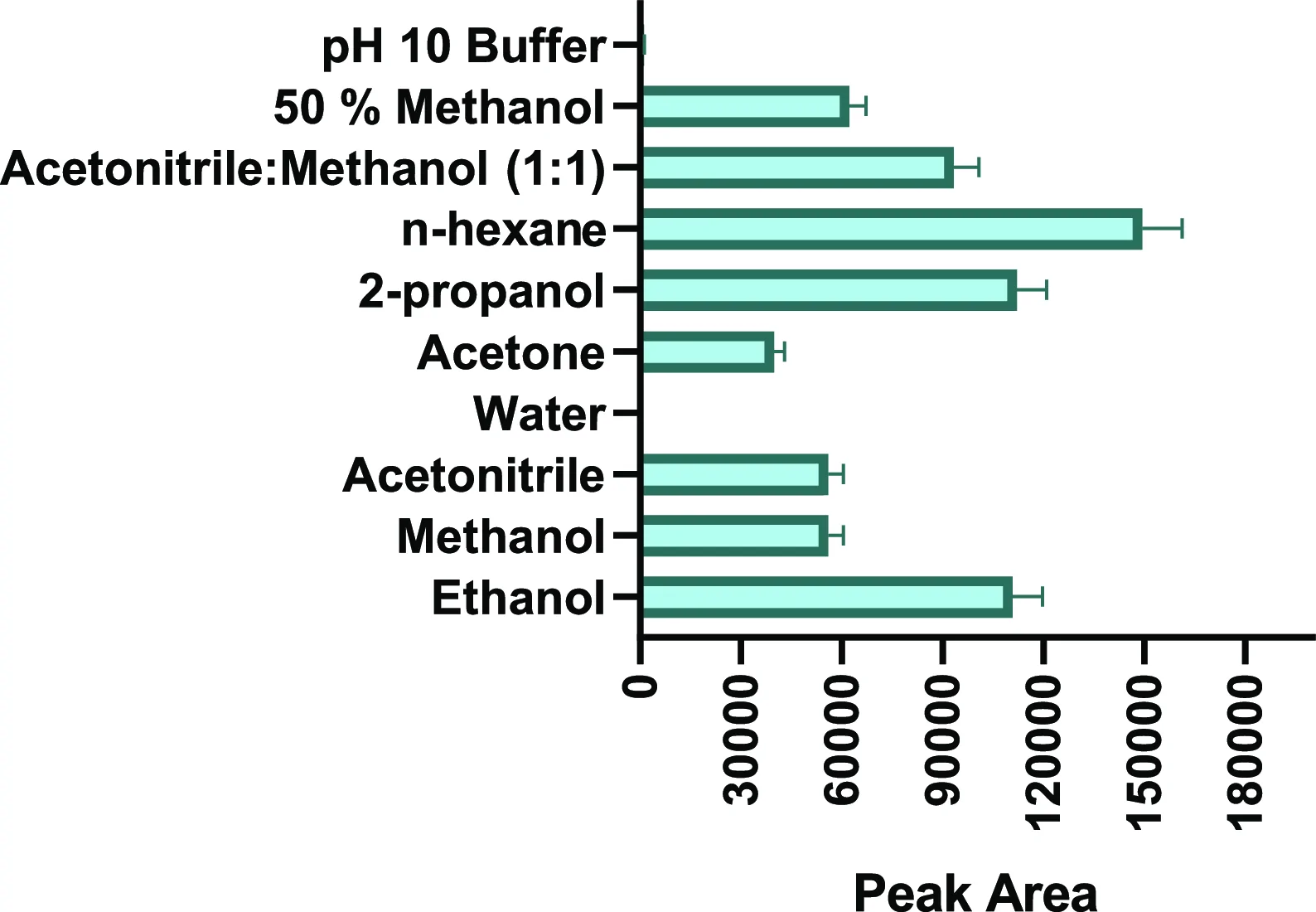
Effect of solvent type for the desorption process by means of model solutions with 250 ng mL^–1^ of duloxetine (DLX) (N:3).

The volume of desorption solvent used to elute duloxetine from the solid phase is a critical factor in preconcentration experiments, as it directly influences the enrichment efficiency. Following the selection of the desorption solvent, the enrichment process was conducted in 10 tubes under identical experimental conditions, employing varying volumes of *n*-hexane ranging from 200 to 1500 μL to determine the optimal conditions for achieving maximum analytical signals.

The results of this optimization (Figure 9) showed that the best elution condition was achieved using 500 μL of desorption solvent (*n*-hexane). Increasing the solvent volume led to a decrease in analyte concentration in the final solution, resulting in a more diluted solution and a corresponding reduction in signal intensity.

**Figure 9:**
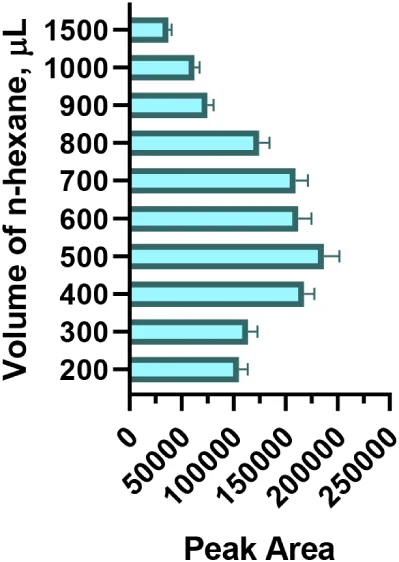
Effect of the quantity of the desorption solvent on magnetic solid-phase extraction (MSPE) evaluated using model solutions with 250 ng mL^–1^ of duloxetine (DLX) (N:3).

Preferably, the MSPE technique should produce quantitative results in the shortest possible amount of time. To determine the optimal vortex time for effective desorption of the analyte into the solvent, experiments were conducted using 10 separate tubes with identical enrichment parameters, varying the vortex time from 0 to 90 s. The results showed that the desorption equilibrium between the adsorbent and the elution solvent was attained in 40 s. The elution efficiency remained relatively unchanged after 40 s. Therefore, for the subsequent experiments, target molecules were desorbed within 40 s of vortex mixing, and the MSPE procedures were performed using the above conditions.

### Reusability and Carry-Over Effect of Developed Magnetic Material

The core performance indicators of the analytical assay—including its selectivity, sensitivity, throughput, robustness, and financial viability—are heavily dependent on the structural features of the engineered magnetic particles. For solid-phase extraction materials, evaluating the carry-over risk and recycling capacity is of paramount importance. To test the reusability profiles of the newly synthesized substrate, standard solutions spiked with 200 ng mL^–1^ of DLX were monitored over ten sequential extraction-desorption cycles using the proposed MSPE protocol. Sorbent regeneration between consecutive runs was achieved by washing the material with 2 mL of an acetonitrile–methanol blend (1:1, v/v) and subsequently drying it under vacuum at 50 °C.

The evaluation of sorbent stability was performed by tracking the chromatographic peak area of DLX across the matrix cycles, calculating relative standard deviation (RSD) trends relative to the initial run. Remarkably, the fluctuation in the analyte’s peak response did not exceed 5.0% RSD even after ten reuses, as documented in Figure 10. These data clearly confirm that the core magnetic responses and surface binding affinities of the nanosorbent remain remarkably stable throughout extended operation. Such excellent recycling efficiency highlights the material’s viability for routine, long-term applications, offering an economically attractive and sustainable tool for high-throughput MSPE frameworks.

**Figure 10:**
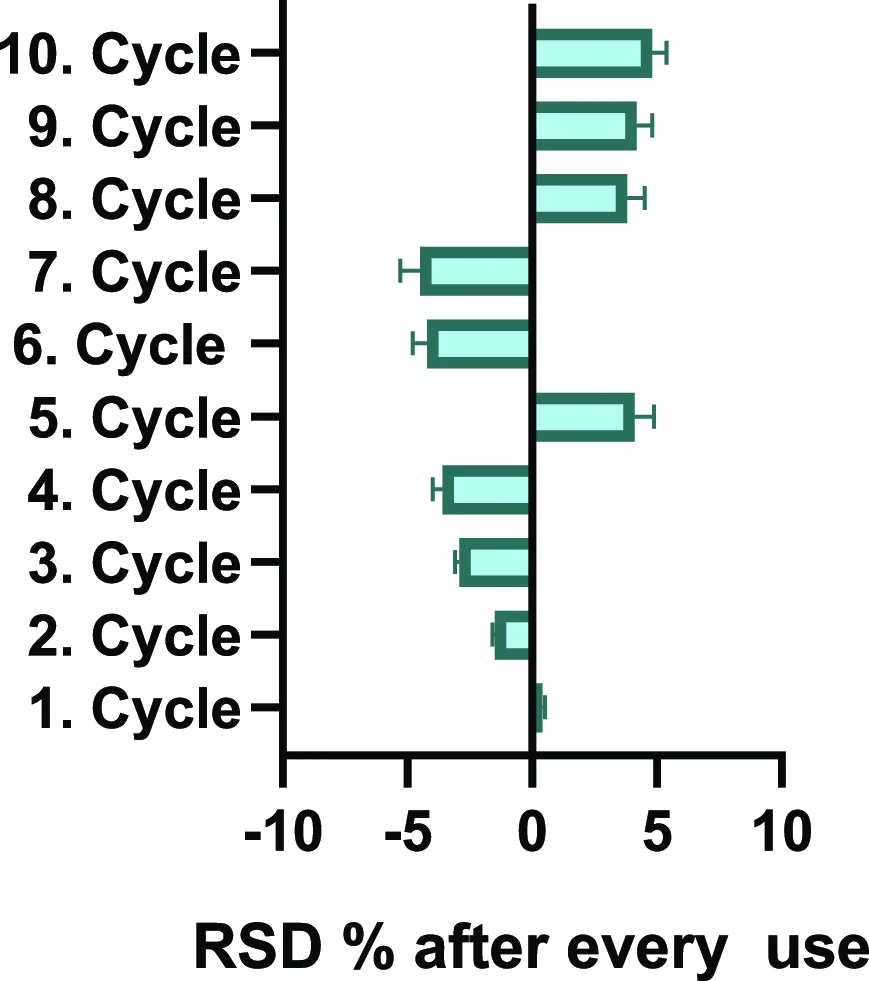
Reusability of the new magnetic sorbent by means of model solutions including 200 ng mL^–1^ of duloxetine (DLX) (N:3).

The carry-over effect was also studied using a model solution containing 200 ng mL^–1^ of duloxetine molecules. For this study, the developed MSPE method was applied to 5 repetitive model solutions under optimized conditions, with each solution interacting with the adsorbent. After every desorption process, the residue of the target molecule was extracted again with the same amount of solvent. This cycle was repeated 3 times. The evaluation of the analytical signal was performed by measuring the peak areas of the duloxetine molecule. As a result, it was observed that the residue of the target molecule on the surface of the magnetic particles after desorption was less than 5% of the total amount of the molecule. In other words, more than 95% of the target molecule can be desorbed with the solvent, ensuring it is not transferred to the next use (carry-over effect).

### Analytical Merits of the Proposed Analytical Method

Following the systematic optimization of all MSPE variables, a comprehensive method validation framework was executed in compliance with the International Council for Harmonization (ICH) technical directives.^46^ The parameters under scrutiny encompassed linearity, accuracy (evaluated via recovery studies), precision (reported as % RSD), limit of detection (LOD), and limit of quantification (LOQ). The validation performance metrics obtained across a realistic concentration range of duloxetine are summarized in Table 1. While the core HPLC-DAD quantification conditions had been preestablished, a baseline validation was initially performed using standard reference solutions. Comparative assessments of linearity, LOD, LOQ, and correlation coefficients (R^2^) were conducted prior to and after the integration of the MSPE step. Notably, implementing the MSPE protocol lowered the sensitivity thresholds (LOD and LOQ) from microgram scales down to nanograms per milliliter levels.

**Table 1:** Analytical Merits of the Established Method

**Parameter**	**Before MSPE**	**After MSPE**
Linearity	1.00–20.00 μg mL^–1^	10.00–800.00 ng mL^–1^
LOD	0.34 μg mL^–1^	3.28 ng mL^–1^
LOQ	0.88 μg mL^–1^	9.60 ng mL^–1^
RSD (%)	6.95 (for 5.0 μg mL^–1^)	5.10 (for 100.0 ng mL^–1^)
Slope	12.54	1141.10
Correlation coefficient (R^2^)	0.9998	0.9942
Preconcentration factor^a^	-	100
Enhancement factor^b^	-	91

a The preconcentration factor (PF) is defined as the proportion of the first solution volume (50 mL) to the final elution solvent volume (0.5 mL).

b The enhancement factors (EFs) were identified by comparing the slopes of the calibration curve of the analytes before and after magnetic solid-phase extraction (MSPE) application.

To verify the practical scope of the assay under optimized conditions, linear calibration functions were constructed using matrix-spiked standards across increasing duloxetine levels. The standard calibration curves were generated by correlating the chromatographic peak areas against the nominal concentrations, allowing the direct calculation of the slopes, intercepts, and regression tracking. Operationally, LOD represents the minimum analyte concentration detectable with acceptable statistical certainty, whereas LOQ defines the lowest concentration boundary that allows reliable quantitative determination with proven precision and trueness. These performance markers were derived directly from the calibration regression equations,^39,40^ utilizing the responses 3.3 σ/S and 10 σ/S, where σ indicates the standard deviation of the regression intercept and S signifies the slope of the corresponding calibration line.

Comparisons of the results obtained before and after MSPE are shown in Table 1. The most prominent features of the developed method are the improvements made in the linear working range and limits of determination. These values are considerably lower than those obtained before application of the MSPE technique.

### Application of the Developed Method in Real Samples

To evaluate the practical applicability of the developed methodology, validation protocols were performed using both human urine matrices and prepared synthetic urine environments. Real urine specimens were obtained from a healthy volunteer who had restricted medication intake and provided explicit informed consent prior to enrollment; the collected samples were drawn into sealed tubes and processed immediately. The synthetic urine substitute was formulated in accordance with established literature protocols.^47^ To achieve this, a salt matrix comprising urea (6.25 g), CaCl_2_·2H_2_O (0.27 g), NH_4_Cl (0.25 g), KCl (0.4 g), Na_2_SO_4_ (0.35 g), KH_2_PO_4_ (0.35 g), and NaCl (0.73 g) was dissolved in 250 mL of deionized water. The solution’s pH was carefully adjusted to 6.0 using a dilute 0.1 M HCl solution, followed by transfer to an amber glass bottle for storage at 4 °C.

Spiking experiments were conducted at three distinct concentration thresholds (100, 250, and 500 ng/mL) by introducing standard DLX solutions into both the real and artificial urine systems independently to complete the recovery assessments (Table 2). The recorded recovery values ranged between 98.1% and 102.2%, paired with precise relative standard deviations of below 5.3%. As demonstrated by the typical chromatogram shown in Figure 11, the analysis of blank urine control matrices revealed no target interference near the retention time of DLX, which strongly confirms the excellent selectivity and specificity of the analytical assay.

**Figure 11:**
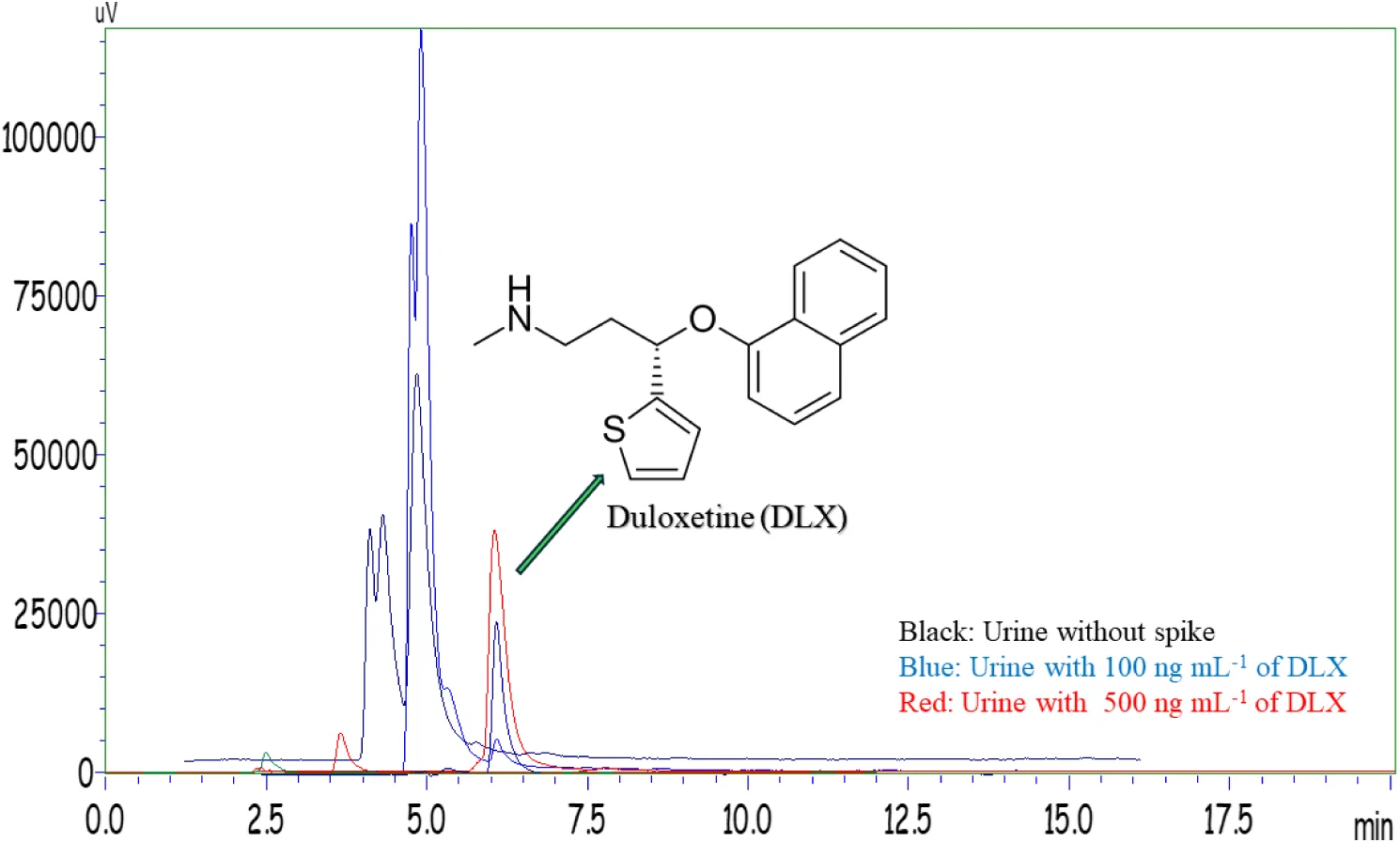
Analysis of urine samples at three spiking levels of duloxetine (DLX) (100, 250, and 500 ng mL^–1^) by using the developed method.

**Table 2:** Application of the Proposed Method to Samples of Human and Synthetic Urine (N:3)

**Sample**	**Added** ng/mL	Found^a^ ng/mL	**RSD** ^b^ **%**	**Recovery** ^c^ **%**
Synthetic urine	0	<LOD	-	-
	100	101.2 ± 3.9	3.9	101.2
	250	253.4 ± 13.5	5.3	101.4
	500	503.4 ± 25.4	5.0	100.7
Human urine	0	<LOD	-	-
	100	102.2 ± 3.4	3.3	102.2
	250	245.3 ± 10.2	4.2	98.1
	500	491.2 ± 23.5	4.8	98.2

a Mean ± standard deviation.

b Relative Standard Deviation (RSD %).

c Recovery values were calculated by means of found and added concentrations (R % = Found/Added × 100).

A comparison of the proposed method with some published methods is presented in Table 3. The proposed HPLC-DAD method demonstrates distinct advantages compared to other analytical approaches listed in the table. The use of MSPE offers higher selectivity in sample preparation compared to simpler techniques such as UV–vis spectrophotometry^42^ or direct HPLC analysis,^44^ which require no pretreatment. In terms of linear range, our method (10.00–800.00 ng/mL) covers a broader concentration span than HPLC-FLD/SPME^45^ (2.5–200 ng/mL) and HPLC-UV/SPE^24^ (2–200 ng/mL), enabling reliable quantification at both low and high concentrations. Regarding the limit of detection (LOD), while ultrasensitive techniques such as LC–MS/MS^22^ and HPLC-FLD^25^ achieve lower values, the 3.3 ng/mL LOD of our method is significantly superior to widely used techniques such as UV–vis^42^ (1.2 μmol/L) and DPV^43^ (0.0059 μmol/L). Furthermore, the HPLC-DAD system offers clear advantages over LC–MS/MS in terms of cost and accessibility, making it more practical for routine analysis. Overall, our method stands out in literature with its wide linear range, reasonable detection limit, selective sample preparation, and cost-effectiveness, making it a strong alternative for various analytical applications.

**Table 3:** Comparison of the Developed Method with Some Previously Published Methods for the Determination of Duloxetine

**Analytical system**	**Sample**	**Sample preparation**	**Linearity**	**LOD**	**Ref.**
High-Performance Liquid Chromatography with Ultraviolet Detection (HPLC-UV)	Plasma samples	Liquid–Liquid Extraction (LLE)	5–2000 ng/mL	5 ng/mL	^1^
High-Performance Liquid Chromatography–Tandem Mass Spectrometry (HPLC–MS/MS)	Rat plasma	-	1.00–200 ng/mL	1.00 ng/mL	^3^
Liquid Chromatography–Tandem Mass Spectrometry (LC–MS/MS)	Human plasma	Solid Phase Extraction (SPE)	0.05–101 ng/mL	-	^20^
Liquid Chromatography–Tandem Mass Spectrometry (LC–MS/MS)	Human plasma	Liquid–Liquid Extraction (LLE)	0.100–100.017 ng/mL	0.04 ng/mL	^22^
High-Performance Liquid Chromatography with Ultraviolet Detection (HPLC-UV)	Human plasma	Solid Phase Extraction (SPE)	2–200 ng/mL	0.7 ng/mL	^24^
High-Performance Liquid Chromatography with Fluorescence Detection (HPLC-FLD)	Pharmaceutical formulation	-	10–600 ng/mL	0.51 ng/mL	^25^
Ultraviolet–Visible Spectrophotometry (UV–VIS)	Pharmaceutical formulation	-	10.000–50.000 ng/mL	400 ng/mL	^42^
Differential Pulse Voltammetry (DPV)	Surface waters	-	10.0–111 ng/mL	1.96 ng/mL	^43^
High-Performance Liquid Chromatography (HPLC)	Urine sample	Directly	0.5–200.000 ng/mL	0.25 ng/mL	^44^
High-Performance Liquid Chromatography with Fluorescence Detection (HPLC-FLD)	Human plasma	Solid Phase Microextraction (SPME)	1–500 ng/mL	0.3 ng/mL	^45^
High-Performance Liquid Chromatography with Diode-Array Detection (HPLC-DAD)	Urine samples	Magnetic Solid Phase Extraction (MSPE)	10.00–800.00 ng/mL	3.3 ng/mL	The proposed method

## Conclusion

In this study, TEOS-riboflavin-coated magnetic nanoparticles were successfully synthesized and applied as an extraction sorbent for MSPE. The use of riboflavin as a surface modifier enabled the development of a functionalized Fe_3_O_4_@TEOS material with improved extraction capabilities. The results demonstrated that the sorbent’s high surface area and rapid mass transfer kinetics, coupled with HPLC-DAD analysis, provided an effective platform for the sensitive and selective detection of DLX at trace levels in human urine. Despite the inherent complexity of biological matrices, the method exhibited high extraction efficiency, excellent sensitivity, a broad linear range, reproducibility, and notable accuracy.

The synthesis and surface functionalization of Fe_3_O_4_@TEOS-riboflavin nanoparticles significantly enhanced both the performance and the environmental sustainability of the method, as evidenced by the reduced use of organic solvents. The overall analytical workflow offers a cost-effective and efficient solution suitable for routine screening and pharmacological investigations involving DLX and related pharmaceuticals. Moreover, the simplicity of the sample preparation process facilitates its application in practical settings. Comprehensive physicochemical characterization techniques, including FTIR and SEM–EDX, confirmed successful surface modification and preservation of the structural integrity of the nanoparticles. The method also achieved high recovery rates with minimal relative standard deviation, further validating the reliability and robustness of the MSPE-coupled HPLC-DAD system for duloxetine analysis. The effective integration of magnetic nanomaterials with modern analytical instrumentation represents a promising direction for advanced drug analysis. By leveraging the benefits of magnetic sorbents and streamlined sample handling, this approach not only enhances analytical precision but also extends the applicability of drug monitoring in diverse and complex sample environments. Ultimately, the findings suggest that the proposed MSPE–HPLC methodology holds significant potential for detecting duloxetine in real samples and offers valuable insights into the broader use of magnetic nanomaterials in separation science

## Data Availability

The data that validate and support the outcomes of this research are integrated directly into the manuscript. Any additional raw datasets or alternative formatting options can be provided by the corresponding author following a reasonable request.
